# Case report: Short rib polydactyly syndrome - type 2 (Majewski syndrome)

**DOI:** 10.4103/0971-3026.63044

**Published:** 2010-05

**Authors:** Pramod Setty Jutur, Chandan Pramod Kumar, Shetteppa Goroshi

**Affiliations:** Department of Radio Diagnosis, J. J. M. Medical College, Davangere - 577 004, Karnataka, India

**Keywords:** Majewski syndrome, short rib polydactyly syndrome type 2, short ovoid tibia

## Abstract

Short rib polydactyly syndrome (SRPS) type 2 (Majewski syndrome) is a rare inherited, autosomal recessive, lethal skeletal dysplasia characterized by horizontally located short ribs, pre- and postaxial polysyndactyly, and micromelia, with characteristic short ovoid tibiae. There may or may not be visceral involvement. We report a case of SRPS type 2 that was diagnosed by antenatal USG at 28 weeks of gestation; the diagnosis was subsequently confirmed by postnatal radiography, fetal autopsy, and histopathology.

## Introduction

Short rib polydactyly syndrome (SRPS) is a rare inherited, autosomal recessive, lethal skeletal dysplasia that can be diagnosed by prenatal USG. It is characterized by micromelia, short ribs, hypoplastic thorax, polydactyly (pre- and postaxial), and multiple anomalies of major organs. There are four types.[1] Here we report a case of SRPS type 2 (Majewski syndrome).

## Case Report

**Figure 1:**
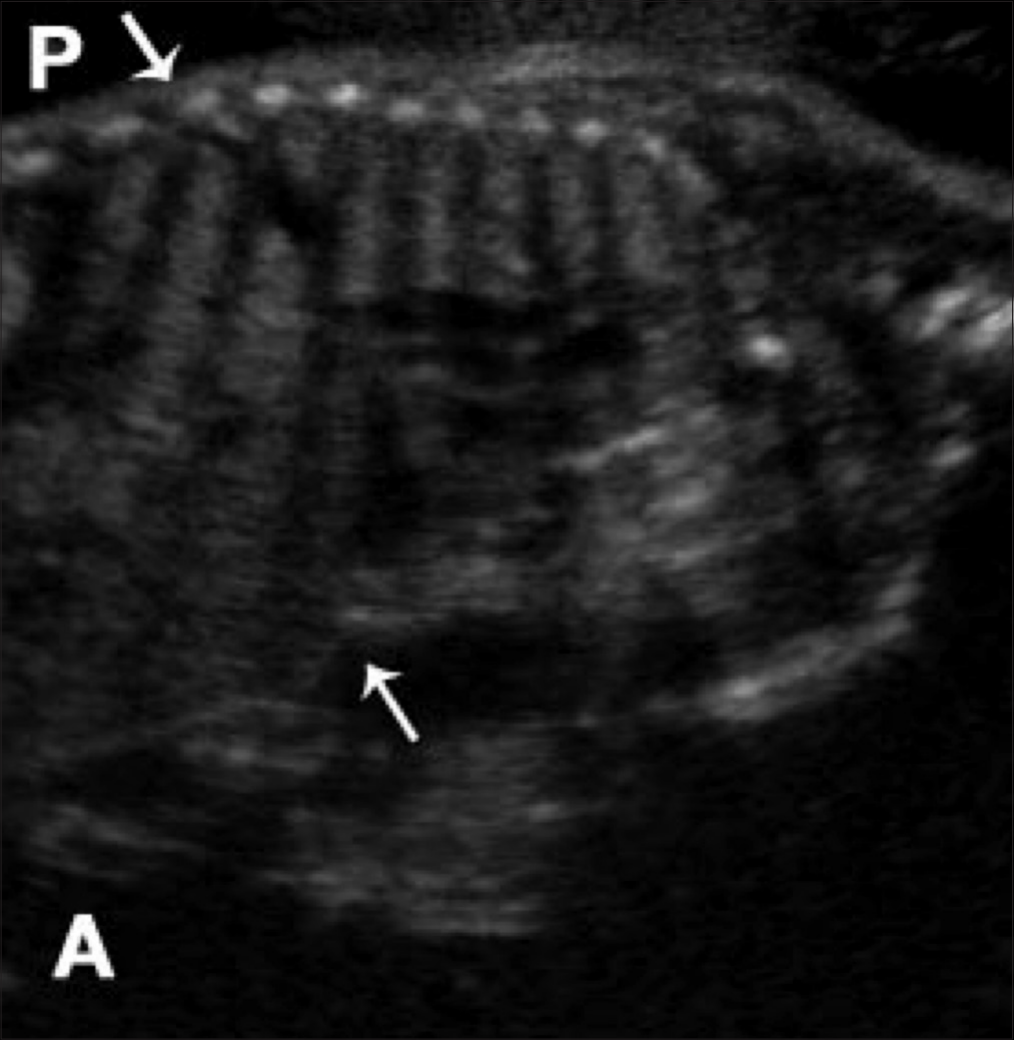
Sagittal USG through the fetal thorax and abdomen shows a narrow thorax (arrows). P, posterior; A, anterior

**Figure 2:**
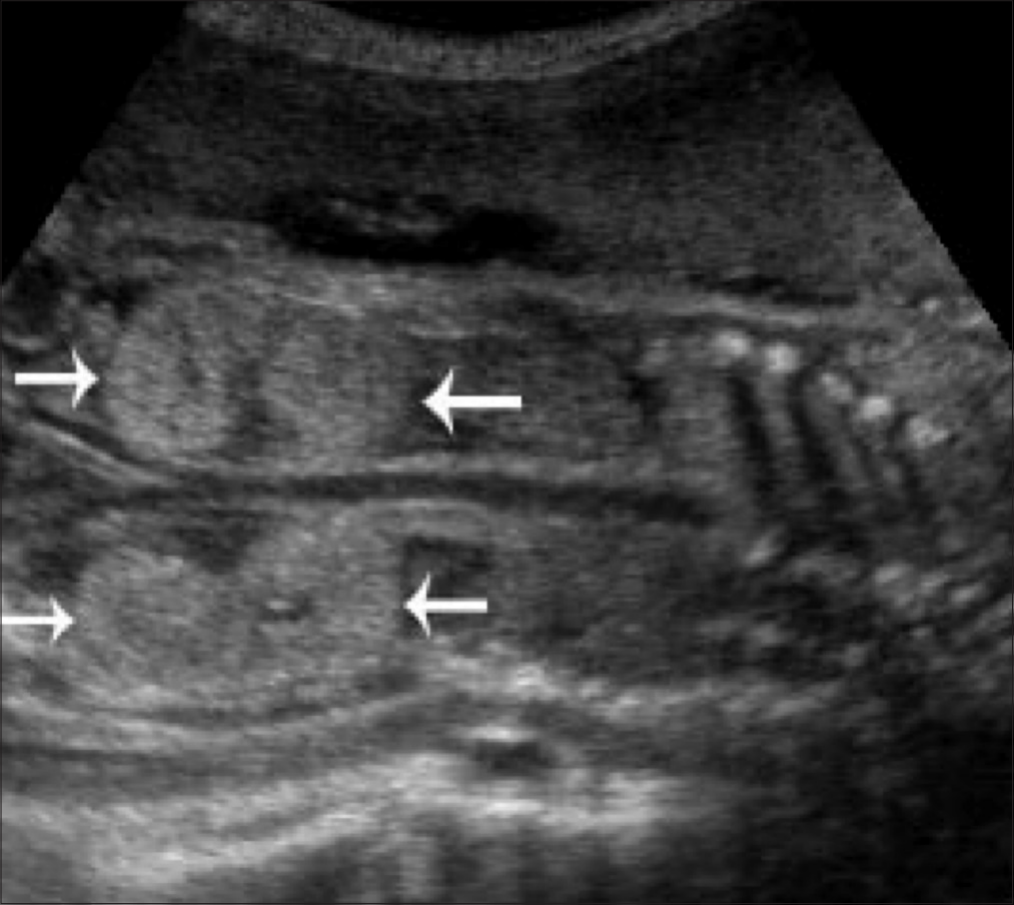
Coronal USG of the fetal kidneys shows bilateral echogenic enlarged kidneys (arrows)

**Figure 3:**
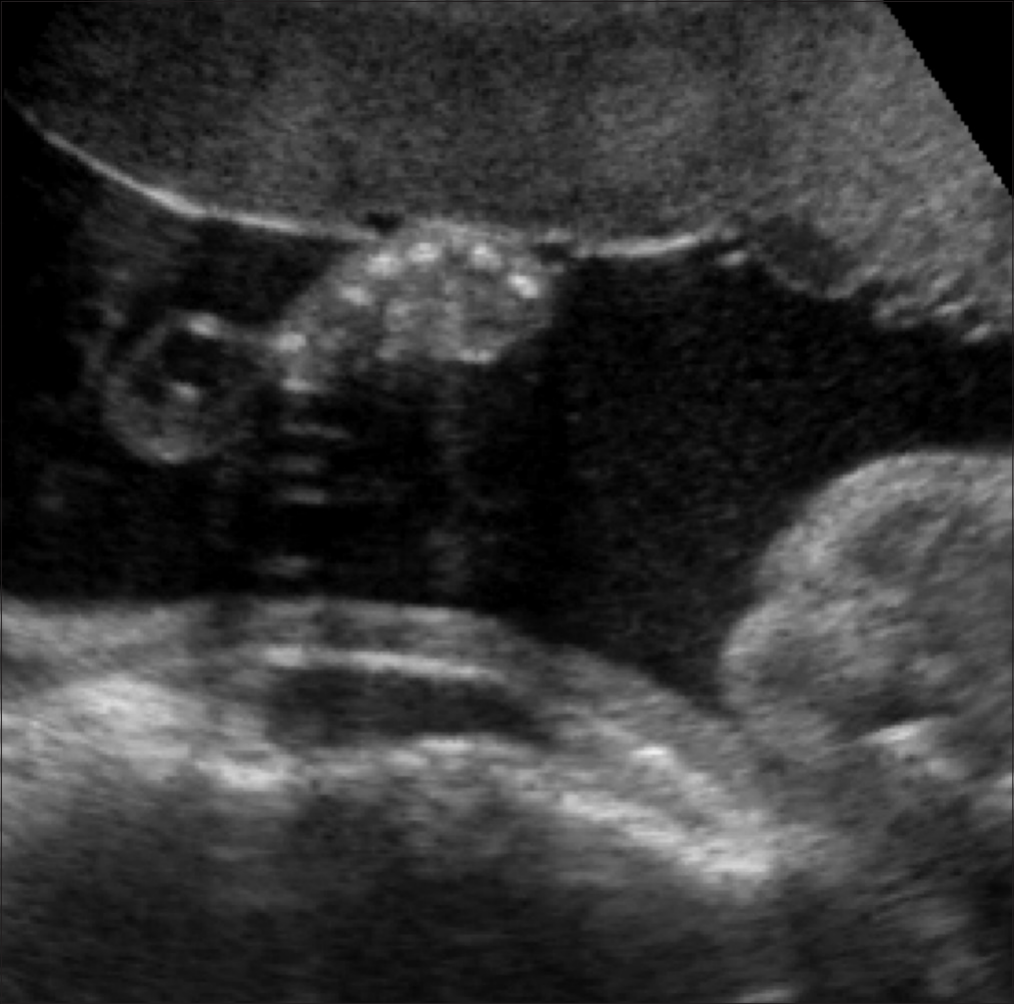
Transverse USG through the hand shows polydactyly

**Figure 4:**
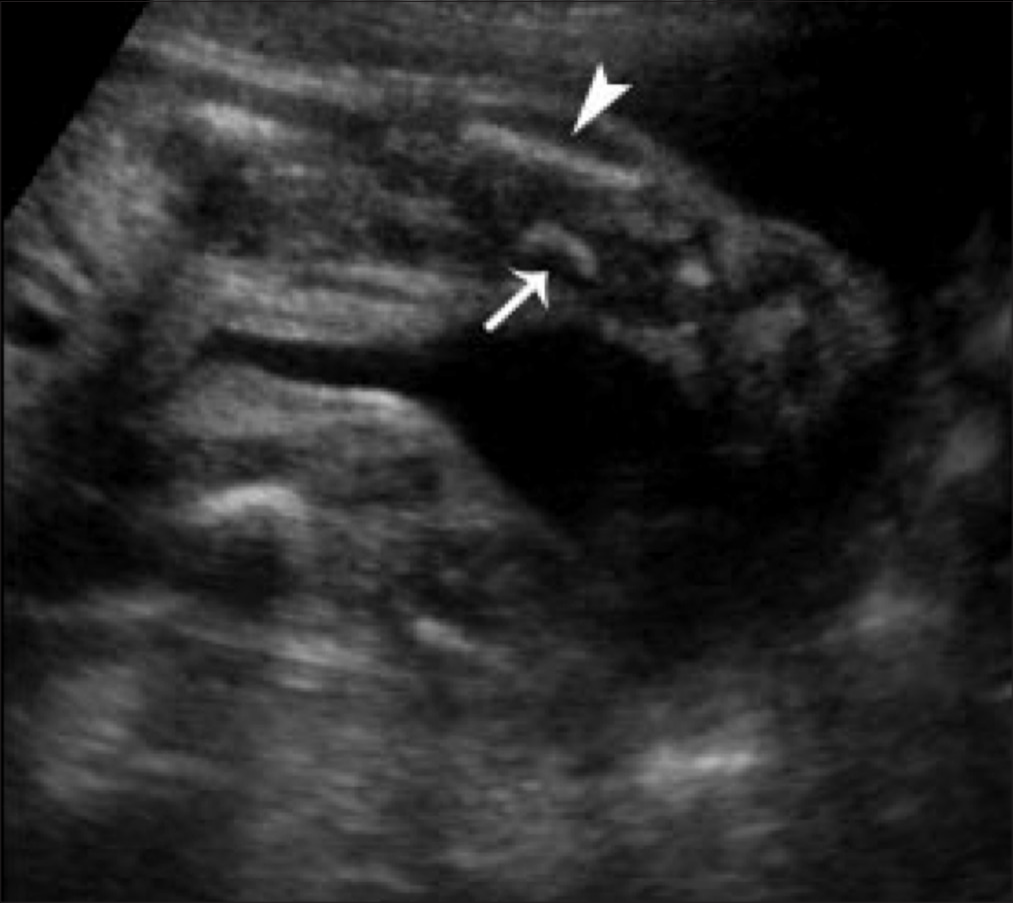
Oblique coronal USG of the left leg shows a short fibula (arrowhead) and an extremely short tibia (arrow)

A 23-year-old woman, who had a second-degree consanguineous marriage, was referred for routine obstetric USG during her last trimester. She was a second gravida with one living child; her previous child was normal. USG revealed a single live intrauterine fetus of 28 weeks gestation (as assessed by biparietal diameter and head circumference); there was also mild polyhydramnios. The fetal thorax was extremely narrow [Figure 1]. Thoracic to abdominal circumference ratio was 0.76 (normal range: 0.77–1.01). USG of the fetal abdomen revealed bilateral enlarged (36 mm) echogenic kidneys and minimal ascites [Figure 2]. The fetal face was hydropic, with indentation of the upper lip. The upper limb bones were micromelic (18-19 weeks gestation) and there was polydactyly [Figure 3]. The lower limb bones were micromelic (19–19.5 weeks), with unusually short tibiae [Figure 4]. The left foot showed hallux varus deformity. Bilateral club feet with polydactyly was also noted. There were no neural tube defects, and the fetal stomach and urinary bladder were normal. Fetal echocardiography revealed no abnormality. The umbilical cord showed a Wharton jelly cyst.

Based on these findings, we arrived at a diagnosis of a lethal skeletal dysplasia, i.e., SRPS type 2. Since this is a lethal disorder, we advised elective termination of the pregnancy followed by fetal autopsy and genetic counseling.

**Figure 5:**
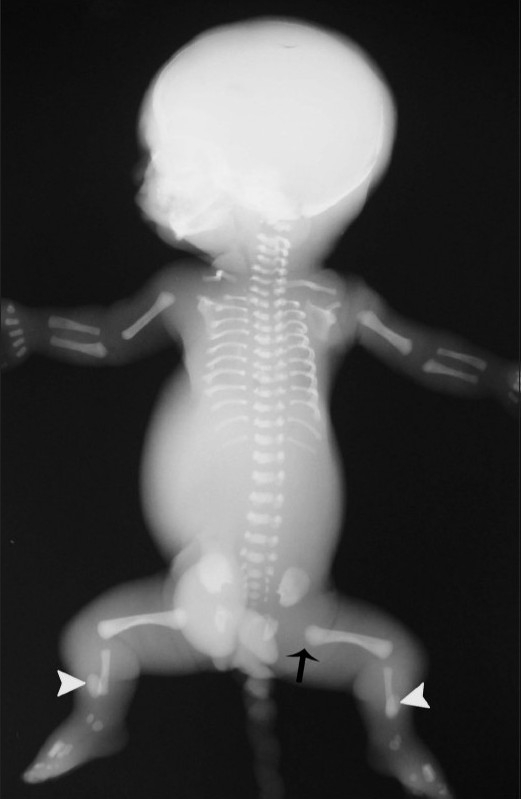
Infantogram after birth shows short and horizontally located 11 pairs of ribs, with a narrow thorax and short upper and lower limb long tubular bones. The metaphyseal ends of the long bones (arrow) are rounded and there are extremely short ovoid tibiae (arrowhead)

Labor was induced and the patient was delivered a female stillborn baby vaginally. The birth weight was 650 g. Infantogram of the stillborn baby [Figure 5] revealed 11 pairs of ribs that were short and horizontally located, a narrow thorax, short upper limb bones, normal iliac bones, short lower limb bones (femur and fibula), extremely short ovoid tibiae, and rounded metaphyseal ends of long bones.

**Figure 6:**
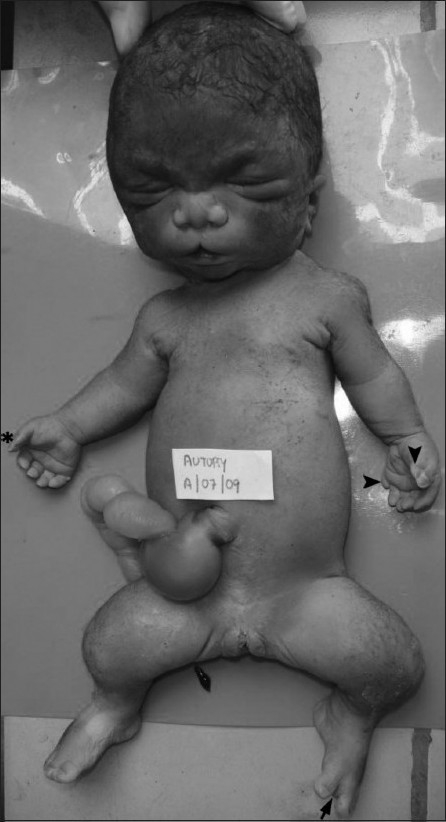
Postnatal photograph of the fetus shows a hydropic face, indentation of the upper lip, broad and flattened nose, depressed nasal bridge, micrognathia, narrow thorax, protuberant abdomen, and Wharton jelly umbilical cord cyst. The right hand shows preaxial polysyndactyly (asterisk) and there is pre- and postaxial polysyndactyly on the left side (arrowhead). There is preaxial polydactyly of the right foot and hallux varus deformity (arrow) of the left foot. Also, note the equino varus deformity of both feet

**Figure 7:**
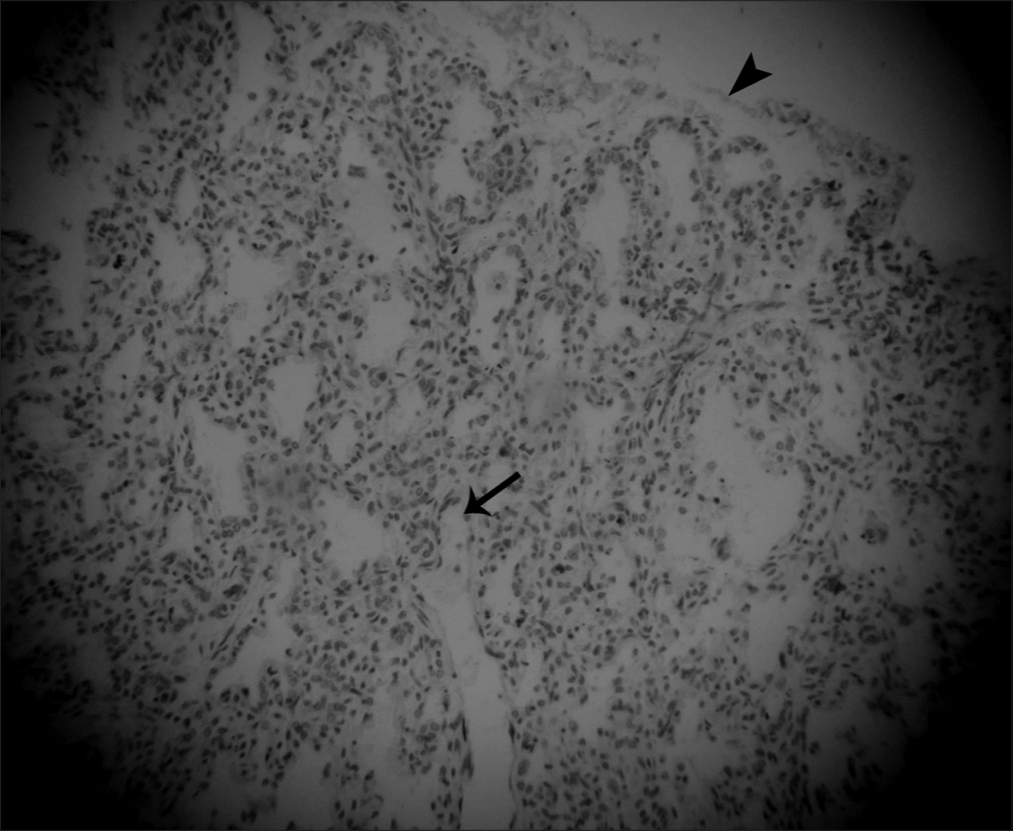
Histopathology section of the lung shows less than four alveolar spaces between the terminal bronchioles (arrow) and the pleura (arrowhead) – suggestive of hypoplastic lung (H and E, ×10)

The gross autopsy findings [Figure 6], such as the bell-shaped thorax, micromelia, pre- and postaxial polysyndactyly, and the clefting of upper lip, along with the histopathological findings [Figure 7] of hypoplastic lungs, hepatic fibrosis with bile duct proliferation, medullary sponge kidneys, and markedly retarded growth zone of femur, supported our prenatal USG diagnosis of SRPS type 2.

## Discussion

SRPS are a heterogeneous group of disorders inherited as an autosomal recessive trait. There is a 25% risk of recurrence in further pregnancies. Traditionally, four major types are recognized: type 1 (Saldino-Noonan), type 2 (Majewski), type 3 (Verma-Naumoff), and type 4 (Beemar-Langer). These conditions are classified within the family of short rib dysplasias with or without polydactyly, a group which includes asphyxiating thoracic dysplasia (Jeune syndrome), and chondroectodermal dysplasia (Ellis-van Creveld dysplasia). SRPS types 1 through 4 are lethal in the newborn period because of the severe pulmonary hypoplasia and the other associated anomalies.[1] On the other hand, the Ellis-van Creveld and Jeune syndromes are not uniformly lethal. Accurate prenatal diagnosis is important in order to provide adequate counseling.[1] SRPS shows female predominance.[2]

Type 2 SRPS (Majewski syndrome) was first described in 1971.[3] The exact incidence is not known but, till 1994, about 33 cases have been reported.[4] The molecular basis of SRPS has not been elucidated. Urioste recognized a balanced pericentric inversion of chromosome 4 in a proband with clinical and radiological manifestations of SRPS and proposed that the disorder could be due to disruption of the gene 4p16 region.[5] Additional chromosomal material may be seen at 17p11 on high-resolution prometaphase analysis.[4]

The clinical manifestations in the fetus are (a) hydropic appearance at birth; (b) facial features of prominent forehead, low-set and malformed ears, lobulated tongue, micrognathia, cleft lip/palate, and a short and flat nose; (c) extremely short and narrow thorax, with a protuberant abdomen; and (d) micromelia (particularly distally), with preaxial and/or postaxial polysyndactyly, brachydactyly, and hypoplasia or aplasia of nails.[4,6] Other reported anomalies include dry skin, cystic kidneys, genital anomalies, pancreatic fibrosis, gastrointestinal tract and brain anomalies (arhinencephaly, vermis hypoplasia, arachnoid cyst, cerebral dysgenesis), hypoplastic epiglottis, larynx and cardiovascular anomalies (atrial septal defects). Death occurs in the perinatal period.[4]

The radiological manifestations include (a) underdeveloped mandible, with irregular teeth; (b) extremely short and horizontally located ribs; (c) limb abnormalities such as mesomelia, with marked shortening of tubular bones (the tibiae are particularly extremely short and have an ovoid configuration), rounded metaphyseal ends of long tubular bones, precocious ossification of proximal femoral epiphysis, polydactyly, distal phalangeal hypoplasia, and symphalangism; and (d) almost normal pelvis.[4]

Prenatal diagnosis by USG is based on the findings of short long bones, very short ribs (narrow thorax), normal vertebrae, polydactyly, hyperextension of head and neck, and large echogenic kidneys.[4] Other anomalies that may be detected by USG include congenital heart disease, median cleft lip, and anophthalmia.[7] On pathological examination, the physis is markedly retarded and disorganized.[8]

SRPS should be differentiated from other skeletal dysplasias presenting with micromelia and thoracic hypoplasia namely achondrogenesis, thanatophoric dysplasia, hypophosphatasia, and osteogenesis imperfecta type 2. However, postaxial polydactyly is present only in SRPS, while hypomineralization is only rarely present in some subtypes of SRPS. Another condition to be distinguished from SRPS is chondroectodermal dysplasia (Ellis-van Creveld syndrome), which too has features of thoracic hypoplasia and postaxial polydactyly; however, in the latter disorder, the thoracic hypoplasia is less pronounced and the limbs are less affected. The occurrence of a median cleft lip identifies type 2 and type 4 SRPS.[7] However, short ovoid tibia are not seen in type 4 SRPS; this is the diagnostic finding of type 2 SRPS (Majewski).[9] The other differential diagnosis are shown in Table 1.

**Table 1:** Disorders with thoracic dysplasia and polydactyly[1],[10]

	Asphyxiating thoracic dysplasia (Jeune)	Chondroectodermal dysplasia (Ellis-van Creveld)	Short rib polydactyly syndrome type 1 (Saldino-Noonan)	Short rib polydactyly syndrome type 2 (Majewski)	Short rib polydactyly syndrome type 3 (Naumoff)	Short rib–polydactyly syndrome Type 4 (Beemer- Langer)
Relative prevalence	Common	Uncommon	Common	Extremely rare	Rare	Rare
Clinical features						
Thoracic constriction	++	+	+++	+++	+++	+++
Polydactyly	+	++	++	++	++	++
Limb shortening	+	+	+++	+	++	++
Congenital heart disease	−	++	++	++	−	
Other abnormalities	Renal disease	Ectodermal dysplasia	Genitourinary and gastrointestinal anomalies	Cleft lip and palate	Renal abnormality	Cleft lip and palate and genitorurinary and gastrointestinal anomalies
Radiographic features						
Tubular bone shortening	+	+	+++	++	+++	++
Short ovoid tibia	−	−	−	+++	−	−
Distinctive feature in femora	−	−	Pointed ends	−	Marginal spurs	
Short, horizontal ribs	++	++	+++	+++	+++	+++
Vertical shortening of ilia and flat acetabula	++	++	++	−	+	++
Defective ossification of vertebral bodies	−	−	++	−	+	++
Shortening of skull base	−	−	−	−	+	−

+ Not common;

+++ most common;

− absent

There is no long-term outcome for this condition as it is invariably fatal during the neonatal period itself.[11] As regards to obstetric management, when SRPS type 2 is suspected in a pregnancy at risk, the option of termination can be offered regardless of the period of gestation.[12]

In conclusion, this article emphasizes the importance of antenatal diagnosis of lethal skeletal dysplasias (SRPS), as termination of pregnancy is indicated and must be followed by genetic counseling for recurrence risk.
